# Web-based processing of physiological noise in fMRI: addition of the PhysIO toolbox to CBRAIN

**DOI:** 10.3389/fninf.2023.1251023

**Published:** 2023-09-27

**Authors:** Darius Valevicius, Natacha Beck, Lars Kasper, Sergiy Boroday, Johanna Bayer, Pierre Rioux, Bryan Caron, Reza Adalat, Alan C. Evans, Najmeh Khalili-Mahani

**Affiliations:** ^1^McGill Centre for Integrative Neuroscience (MCIN), Ludmer Centre for Neuroinformatics and Mental Health, Montreal Neurological Institute (MNI), McGill University, Montreal, QC, Canada; ^2^BRAIN-TO Lab, Krembil Brain Institute, University Health Network, Toronto, ON, Canada; ^3^Translational Neuromodeling Unit, Institute for Biomedical Engineering, University of Zurich and ETH Zurich, Zurich, Switzerland; ^4^Center for Youth Mental Health, The University of Melbourne, Melbourne, VIC, Australia; ^5^Orygen Youth Health, Orygen, Melbourne, VIC, Australia; ^6^Department of Electrical and Computer Engineering, Concordia University, Montreal, QC, Canada

**Keywords:** neuroimaging, software, fMRI, brain imaging data structure (BIDS), physiological noise correction, high performance computing (HPC)

## Abstract

Neuroimaging research requires sophisticated tools for analyzing complex data, but efficiently leveraging these tools can be a major challenge, especially on large datasets. CBRAIN is a web-based platform designed to simplify the use and accessibility of neuroimaging research tools for large-scale, collaborative studies. In this paper, we describe how CBRAIN’s unique features and infrastructure were leveraged to integrate TAPAS PhysIO, an open-source MATLAB toolbox for physiological noise modeling in fMRI data. This case study highlights three key elements of CBRAIN’s infrastructure that enable streamlined, multimodal tool integration: a user-friendly GUI, a Brain Imaging Data Structure (BIDS) data-entry schema, and convenient in-browser visualization of results. By incorporating PhysIO into CBRAIN, we achieved significant improvements in the speed, ease of use, and scalability of physiological preprocessing. Researchers now have access to a uniform and intuitive interface for analyzing data, which facilitates remote and collaborative evaluation of results. With these improvements, CBRAIN aims to become an essential open-science tool for integrative neuroimaging research, supporting FAIR principles and enabling efficient workflows for complex analysis pipelines.

## 1. Introduction

The preprocessing of fMRI data is often a complex and computationally intensive task. There are several standardized and popular software libraries for typical fMRI analyses [such as SPM (Ashburner et al., 2021), FSL (Jenkinson et al., 2012), AFNI (Cox, 2012), and fMRIPrep (Esteban et al., 2019)], but model-based evaluation of physiological measurements, such as electrocardiogram or breathing belt readings, are not frequently done. This is partly because of the heterogeneity of data formats and lack of standardized methods for their analysis, which creates barriers to prioritizing their treatment. This is despite the fact that the blood oxygen level-dependent signal (BOLD) is strongly influenced by patterns of cardiac and respiratory activity (Birn et al., 2006, 2008; Chang and Glover, 2009; Khalili-Mahani et al., 2013; Murphy et al., 2013). Rhythmic activity of the heart and lungs can cause variations in blood oxygenation, which can be misinterpreted as a marker of neural activity. Respiration can also cause phasic movements of the head and body, as well as pseudomotion caused by field interactions (Power et al., 2019). Model-based physiological image correction has been shown to substantially improve signal-to-noise ratio in task-based fMRI studies (Hutton et al., 2011) and reduce spurious correlations in resting-state networks (Birn, 2012). Khalili-Mahani et al. have also shown that the choice of model in physiological noise reduction affects the inferences that can be made at group-level analyses (Khalili-Mahani et al., 2013).

Accounting for these sources of noise requires iterative model testing, which can take substantial researcher time, expertise, and computational resources (potentially including the purchase and maintenance of costly hardware). Excellent open-source tools exist for efficiently computing these models, such as the MATLAB-based PhysIO toolbox (Kasper et al., 2017), which has been developed to generate various noise models. PhysIO, currently in version 8.0.1, is part of the TAPAS package (https://github.com/translationalneuromodeling/tapas; Frässle et al., 2021), which leverages the Statistical Parametric Mapping (SPM12) library (Friston, 2003; Ashburner et al., 2021). The PhysIO matlab package is one of the most standard and commonly used softwares for model-based physiological noise reduction. It contains a variety of state-of-the-art and commonly used models for model based noise correction in fMRI (Glover et al., 2000; Birn et al., 2008; Chang et al., 2009). A unique feature of this toolbox is that it accepts input from a variety of devices and vendors, and provides an array of modeling options such as RETROICOR (Glover et al., 2000), RVHRCOR (Chang and Glover, 2009), and RVH (Birn et al., 2008); as well as estimated movement data. However, it relies on local installations of MATLAB and SPM, which makes it difficult to use on large or distributed datasets.

Increasingly, neuroimaging researchers rely on open-access shared datasets, containing anywhere from dozens to thousands of subjects (Poldrack and Gorgolewski, 2017; Madan, 2022), such as the Human Connectome Project (Elam et al., 2021), the UK Biobank (Collins, 2012), and the Adolescent Brain Cognitive Development study (Casey et al., 2018). These large datasets, containing hundreds of gigabytes of data, can be infeasible to process on local machines. In addition, comparing the results of different models depends on performing visual or mathematical comparison of pre- and post-corrected datasets, which adds significant time to the workflow. Ideally, a user would want a readily available method of examining the topographical pattern of physiological modulation of the BOLD signal to critically evaluate the significance of confounding effects. To address these challenges, and to facilitate web-based preprocessing for fMRI datasets, we have implemented PhysIO on CBRAIN, an open-source, high performance scientific computing platform.

CBRAIN has been created to address the challenges involved in Big Data research, for example, developing secure and robust ways of leveraging high-performance computing (HPC) clusters for neuroimaging research (https://cbrain.ca/; Sherif et al., 2014). It enables users to launch a large number of tasks, remotely and in parallel, from a user-friendly interface, without the need to install local software or rely on a specific operating system. It is accessed and operated entirely from a web browser and can be used on any major operating system. CBRAIN offers provenance tracking, data management, data visualization, and data sharing features that simplify and accelerate collaborative research. In this paper, we describe the process of integrating PhysIO onto CBRAIN, and illustrate the advantages of incorporating such tools in terms of workflow efficiency and quality-of-life features. The following features have been added to extend the capabilities of PhysIO:

1. PhysIO tasks can run on high-performance servers via a simple graphical user interface, thus alleviating the need of researchers to maintain local servers.
2. Quality-of-life features for researchers to reduce the burden of developing scripts for batch processing, in addition to automated image correction and in-browser visualization of results.
3. Comprehensive provenance tracking enables researchers to track the reproducibility of physiological noise processing in fMRI research.

In sum, this case study highlights the benefits of CBRAIN’s features and infrastructure in supporting multimodal data analysis on large datasets. Furthermore, it serves as a manual for how additional tools may be integrated into CBRAIN’s growing library of image processing software. As an open-source, open-science platform, CBRAIN can be extended and replicated by varied users and research labs, with additional tool integrations potentially coming from members of the neuroimaging community.

## 2. Methods

**Figure 1:**
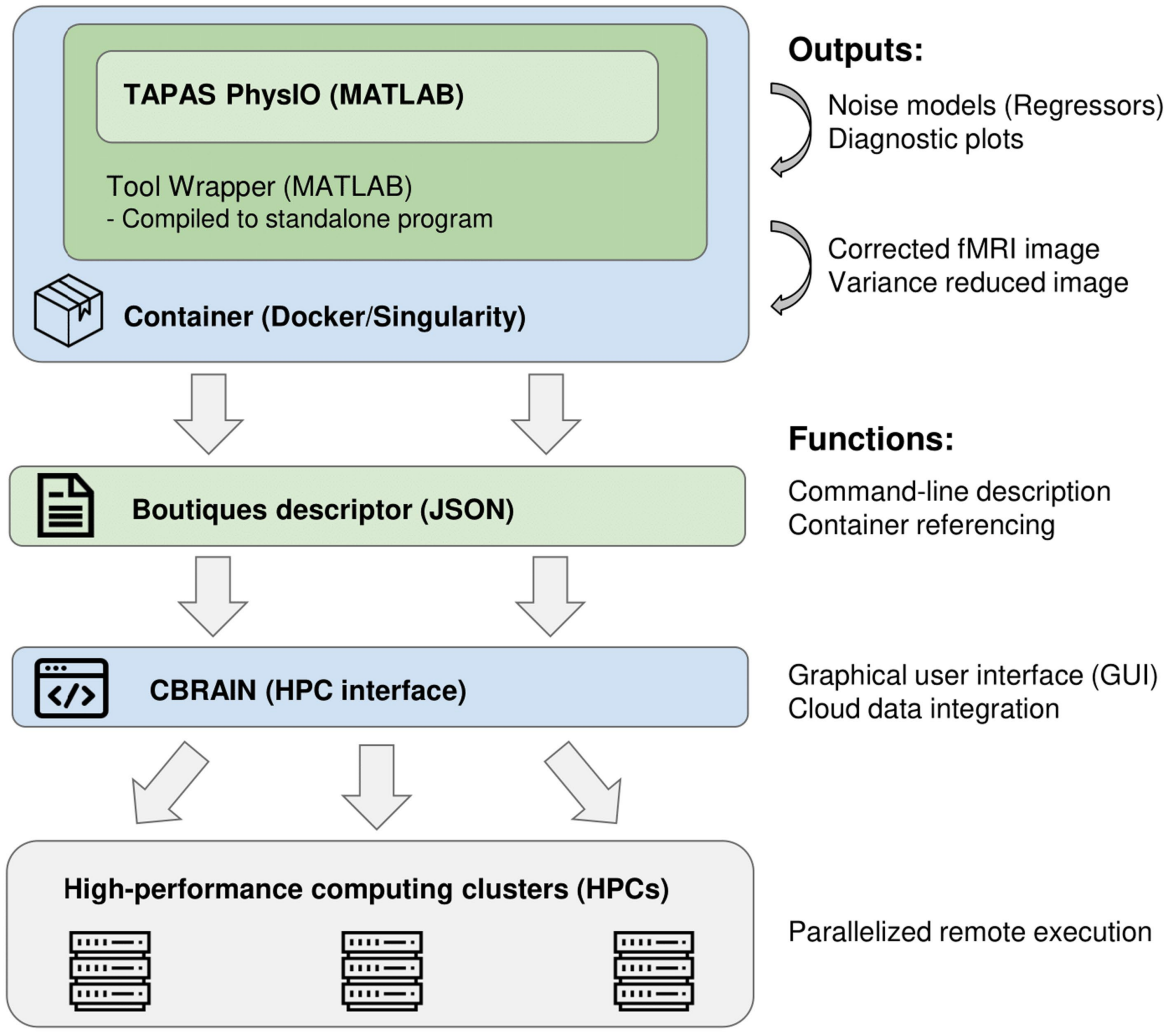
Software pipeline of integration of the PhysIO toolbox into CBRAIN and high-performance computing clusters.

The integration of PhysIO on CBRAIN is accomplished through three main steps (illustrated in Figure 1):

1. The creation of a wrapper program that converts the MATLAB-based script into a command-line tool, and which extends PhysIO with additional functions (e.g., automated image correction, BIDS Subject read-in).
2. Compilation and containerization of the MATLAB application within a Singularity environment (Kurtzer et al., 2017).
3. Creation of a standardized GUI via the Boutiques framework (Glatard et al., 2018).

### 2.1. Wrapper script

To integrate tools into CBRAIN and execute them on high-performance computing servers, the tool must be capable of being invoked and configured entirely through the command-line. For PhysIO, this requires us to write a wrapper script with a command-line interface in MATLAB. This wrapper script also allowed us to add additional functions for automated fMRI image correction, as well as quality-control noise-variance maps based on comparison of corrected and uncorrected images. These additions were programmed in MATLAB 2021b.

#### 2.1.1. Command line parameterization

A command-line interface for PhysIO, which is ordinarily prepared through MATLAB configuration scripts, was written using MATLAB’s inputParser class. The input parameters, numbering some 60–70 arguments, are parsed and loaded into the PhysIO options structure. Ordinarily, these parameters would have to be set by manually editing lines in a MATLAB script. In CBRAIN, the description of this command-line interface using the Boutiques framework (see Section 2.3) allows parameters to be set in a user-friendly online graphical user interface.

#### 2.1.2. Read-in of BIDS data

In recent years, there has been a push to standardize the storage and naming convention of brain imaging data, in order to improve the interoperability and readability of datasets from various research groups and projects. This initiative, the Brain Imaging Data Structure (BIDS) (Gorgolewski et al., 2016), has also been applied to neuroimaging software as the BIDSapps framework (Gorgolewski et al., 2017). The goal of this framework is to leverage the standardized naming schemes and data types of BIDS by configuring preprocessing and analysis scripts to read and process entire subject folders or datasets automatically, rather than having to separately provide the various input files. This greatly increases the speed and ease of neuroimaging analysis pipelines.

In accordance with this framework, we added the capability to read BIDS Subject directories for automatic processing of all the data contained within. Thus, rather than having to separately select an fMRI file and one or more physiological files for every fMRI run to be processed by PhysIO, users on CBRAIN can simply supply a BIDS Subject, which can contain anywhere from one to dozens of separate acquisitions which are read and processed automatically.

To allow users to leverage this BIDS read-in capability, and to facilitate the storage of data in BIDS format more broadly, we developed a prototype utility on CBRAIN for converting datasets from an arbitrary naming and storage convention to BIDS conventions. This tool, “BIDS-Converter,” is written in Python and takes as input wildcards for identifying subject numbers and key-value pairs for identifying and naming files by their modality and other relevant information (BIDS “entities”).

This tool is useful for renaming datasets that have been converted to NIfTI but have not been named and organized in accordance with BIDS conventions. For data that is available in source or DICOM format, users are encouraged to use tools such as dcm2niix (Li et al., 2016) or HeuDiConv (Halchenko et al., 2023) to convert their data from DICOM to NIfTI, which can also automatically organize the outputted files as a BIDS directory complete with all the metadata that is available in the source file headers.

#### 2.1.3. Automated fMRI noise reduction

The main outputs of PhysIO are time-series vectors which can be derived from a variety of well-established models, including RETROICOR (Glover et al., 2000), RVT (Birn et al., 2008), and HRV (Chang et al., 2009). These can be used in a generalized linear model (GLM) to factor out the variance due to cardiac and respiratory activity. In the default implementation of PhysIO, the user is encouraged to do this as a separate step in the Statistical Parametric Mapping (SPM) package in MATLAB (Friston, 2003; Ashburner et al., 2021) and integrate the physiological noise model with any task-based regressors in a unified GLM that includes pre-whitening and high-pass filtering. This avoids over-correcting fluctuations that are correlated with both task and physiology, as well as introducing spurious high-frequency correlations (Bright et al., 2017; Chen et al., 2017). However, it requires multiple user operations within the SPM12 graphical user interface, or custom MATLAB scripting using SPM functions.

We have simplified this task by adding the image correction step to the pipeline, adapted from Chang and Glover (2009), which uses an analytic linear regression step to compute the beta weights of the outputted PhysIO regressors relative to the fMRI data. To compute the beta weights, we use the mldivide operator in MATLAB to solve the system of linear equations X*B = Y, where *X* is the design matrix (including an intercept, time vector, time squared vector, and PhysIO outputs), *Y* is the fMRI BOLD data, and *B* is the beta matrix. The beta-weighted regressors are then subtracted from the original image to produce a noise-corrected fMRI image. Thus, the equation for the correction step is


$$Y_{\mathrm{corrected}}=Y_{\mathrm{uncorrected}}-\left(B^{*}X\right)$$

**Figure 2:**
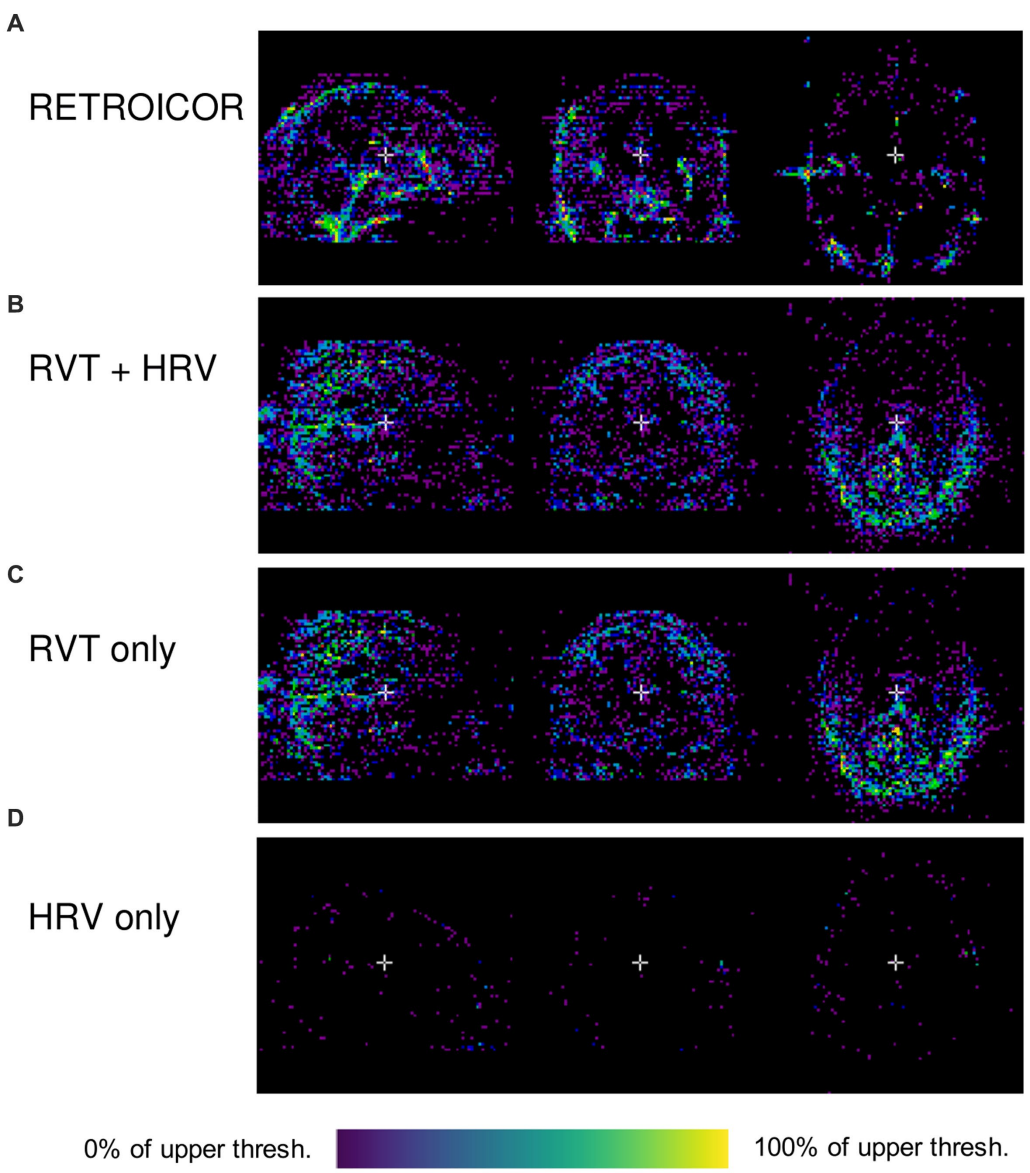
Outputs of PhysIO on CBRAIN (Variance reduced map; pct_var_reduced.nii.gz) using various noise modeling algorithms available in PhysIO: **(A)** RETROICOR (Glover et al., 2000) **(B)** RVT (Birn et al., 2008) along with HRV (Chang et al., 2009), **(C)** RVT alone **(D)** HRV alone. The variance maps were visualized using CBRAIN’s browser-based visualization module. The data used to produce the images are available on OpenNeuro.org (Etzel and Braver, 2020). As the voxels indicate percentage of total variance, the lower threshold in all images is zero; the upper thresholds are 86.6% (0.866) for **(A)**, 19.2% (0.192) for **(B)**, 17.8% for **(C)**, and 18.1% for **(D)**. The images have a lower-bound cutoff of 0.1 for **(A)** and 0.01 for **(B–D)**.

In addition to the corrected image, a three-dimensional pct_var_reduced image is computed where each voxel represents the percentage of variance reduced by noise correction for that voxel’s time series. This computation was also adapted from Chang and Glover (2009). The formula for the variance reduced image is


$$\left(\sigma 2_{\mathrm{uncorrected}}-\sigma 2_{\mathrm{corrected}}\right)/\sigma 2_{\mathrm{uncorrected}}$$

for every voxel in the original image, where *σ2* is the variance of the voxel’s time series. Examples of these variance maps using different noise modeling algorithms available in PhysIO are shown in Figure 2. As it can be seen in this image, the topography of noise variance is dependent on the model, underlining the importance of comprehensive physiological noise modeling in fMRI analyses.

The corrected image produced by this regression modeling step can be used to create visual or qualitative maps of model performance (such as the percent variance reduced image), but the current implementation lacks the prewhitening and high-pass filtering preprocessing steps that would make the output image appropriate for statistical analysis (Bright et al., 2017; Chen et al., 2017). A future update to the PhysIO wrapper on CBRAIN would include these steps, so that the corrected image is ready for hypothesis testing in further task-related GLM modeling, or for assessing the fit of the noise model with statistical tests.

### 2.2. Containerization

Container engines, similar to virtual machines, allow software to be run in a virtual, self-contained computing environment under an operating system which can differ from the host machine’s. This ensures reproducibility and accessibility across multiple platforms and addresses the problem of unstable and changing dependencies in research software. It also allows software pipelines to be easily run on remote high-performance computing servers, such as those leveraged by CBRAIN. Containers are saved and shared as “images,” which are templates that define how a given container will be constructed.

We compiled the PhysIO Toolbox, along with the wrapper script and additional functions, into a standalone application using the MATLAB compiler toolkit. This allows the tool to be run as an executable, without requiring a MATLAB license and installation. Furthermore, the MATLAB compiler toolkit contains functionality for procedurally building a Docker container image with correct dependencies and an appropriate version of the MATLAB runtime binaries. We used this tool to create a Docker image.

While Docker is a widely-used container engine, Docker images are not directly supported on the high-performance computing servers leveraged by CBRAIN. This is because, until recently, Docker Engine required root permissions, which would create security issues on public servers. Singularity (Kurtzer et al., 2017), now called Apptainer, is another open-source container engine specialized for scientific computing. Singularity is natively rootless, i.e., it is optimized for running containers with user privileges, which mitigates security risks, and it provides improved reproducibility for scientific computing (Mitra-Behura et al., 2022). It is interoperable with Docker, as Docker images can be automatically converted to Singularity.

Therefore, at runtime, Boutiques software (see Section 2.3) uses a reference to the Docker image for the PhysIO CBRAIN tool, housed on Docker Hub, and performs the conversion of the image to Singularity. CBRAIN then builds and runs the container on HPC clusters, and caches the Singularity image for future use.

### 2.3. Boutiques descriptor

CBRAIN uses the Boutiques framework (Glatard et al., 2018) to describe and validate command-line inputs. The central component of Boutiques is a JSON descriptor, which contains a dictionary of all the arguments which can be passed to the application. The argument objects include properties such as type, default value, value choices, required or optional, and a text description. The descriptor can also define argument groups, and contains information about the tool version, online repositories, and other metadata.

**Figure 3:**
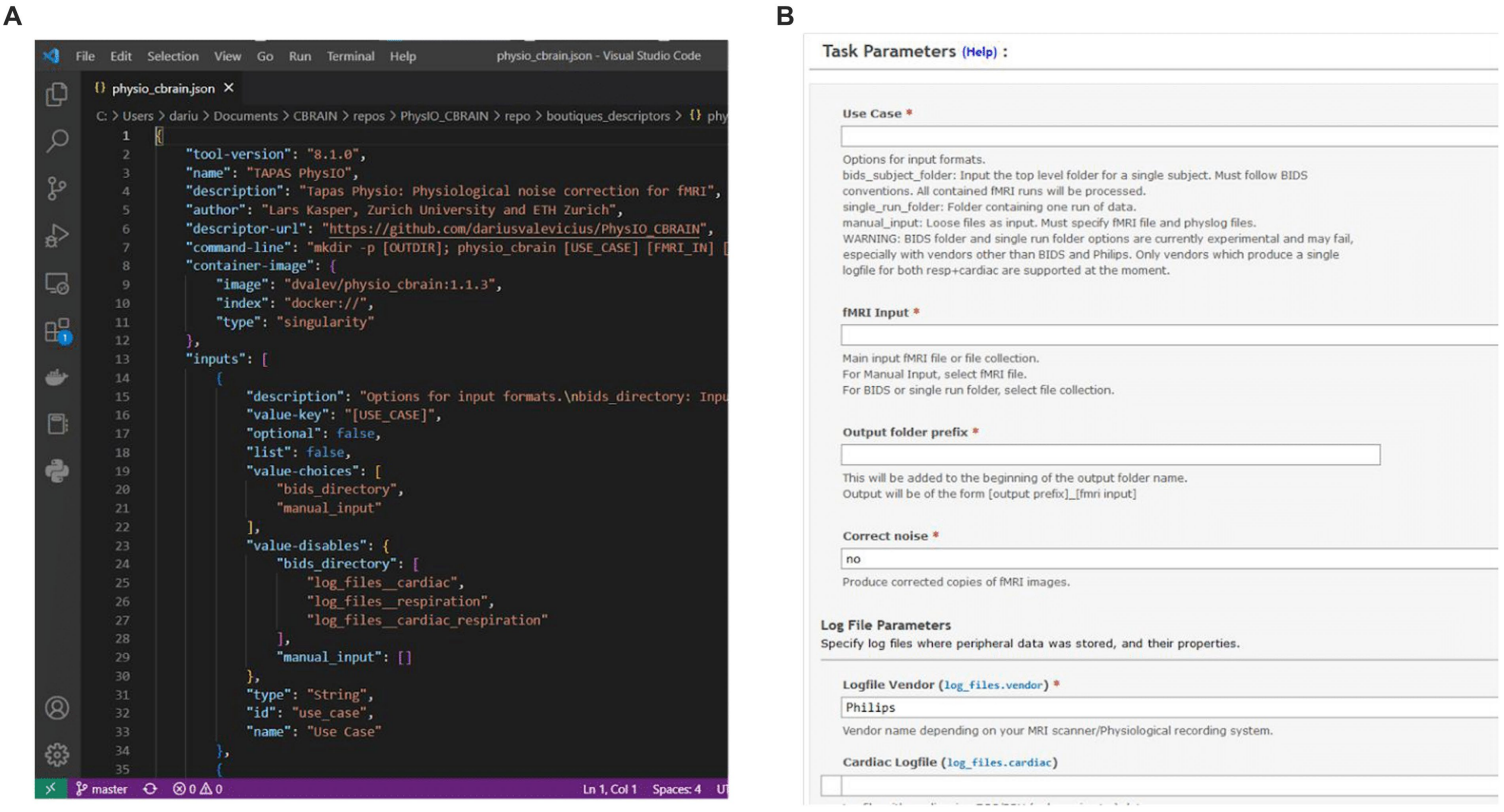
**(A)** Boutiques descriptor JSON specifying information such as the tool version and container address, as well as properties of command-line inputs. **(B)** CBRAIN graphical user interface for task configuration, which is procedurally generated from the Boutiques descriptor.

CBRAIN possesses a streamlined system for tool integration through Boutiques - for any developer to add a new tool to CBRAIN, it is only required to create a container and descriptor for the tool, and to provide the descriptor in a repository on GitHub. The CBRAIN administrators can then integrate the tool (see below). CBRAIN leverages Boutiques to validate task parameters and uses the command-line schema, in combination with a container reference, to launch the tool on high-performance computing clusters. In addition, CBRAIN uses the Boutiques descriptor to populate the GUI for task configuration, thus creating a uniform user interface across different neuroimaging pipelines (Figure 3).

The Boutiques framework is flexible, and while it is leveraged by CBRAIN, it can also be used to configure and run tool containers on local machines. Information on how to integrate Boutiques in local processing pipelines can be found in its online documentation. Boutiques descriptors can furthermore be published on Zenodo (https://zenodo.org/) for online documentation and sharing.

#### 2.3.1. Descriptor integration in CBRAIN

Tool wrappers, executable containers, and Boutiques descriptors are created by tool developers (pipeline contributors) and the workflow and user experience can optionally be mock-tested via local builds of CBRAIN. However, final integration in CBRAIN is performed by designated CBRAIN administrators who will oversee the integrity of the process. Researchers who are interested in getting their tool integrated must contact the administrators either by email or through the CBRAIN support forum. The process is handled on a case by case manner, in order to ensure the compliance of tools with the network’s cybersecurity measures, and to prevent potential malware being installed on the network.

### 2.4. Workflow comparison

To compare the efficiency and functionality of PhysIO in its native MATLAB implementation against the CBRAIN implementation, we used a dataset of 62 fMRI datasets acquired on a Philips 3 Tesla Achieva TX MRI scanner using a 32-channel SENSE head coil (Philips Medical Systems, Best, The Netherlands). Whole-brain fMRI data sets were acquired using T2*-weighted gradient-echo echo-planar imaging with the following scan parameters: 190 volumes; 38 axial slices scanned in ascending order; repetition time (TR) = 2.2 s; echo time (TE) = 30 ms; flip angle = 80°; FOV = 220 × 220 mm; 2.75 mm isotropic voxels with a 0.275 mm slice gap. For all datasets, respiration and cardiac data were acquired with the MR machine’s respiration belt and pulse oximeter, sampled at 500 Hz (Sitsen et al., 2022). We assessed differences between running PhysIO on CBRAIN versus on a “typical student computer” (MSI laptop computer running Windows 10 with an Intel i7-8565U CPU @ 1.80GHz) in terms of user- and computation-time required for each implementation, as well as the functionality provided by each.

**Figure 4:**
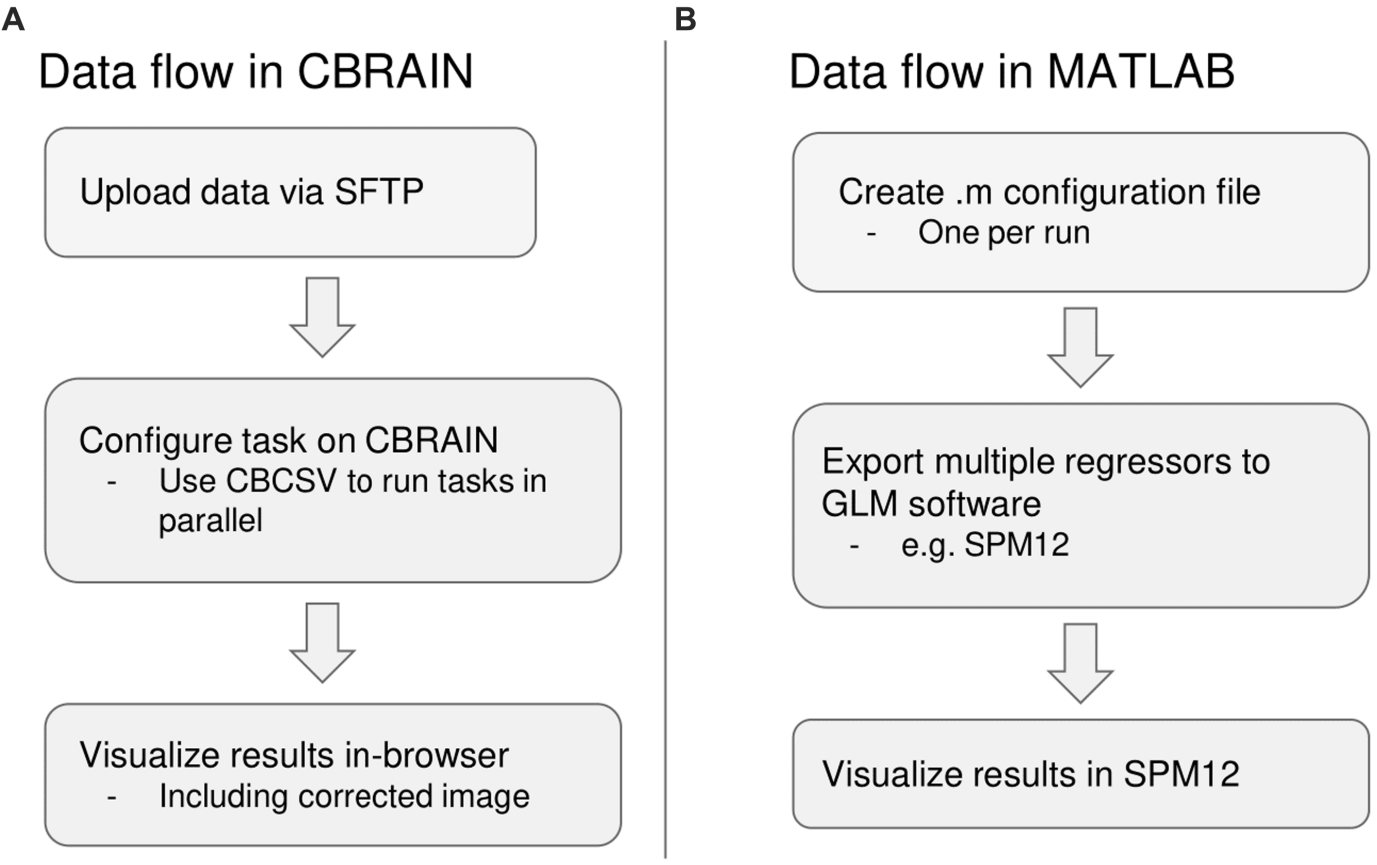
Summary of differences in data flow between CBRAIN and the native MATLAB implementation. **(A)** Data flow in CBRAIN; **(B)** Data flow using native MATLAB implementation. Acronyms: SFTP, Secure File Transfer Protocol (e.g., FileZilla); CBCSV, CBRAIN File List (a comma-separated value file that can be auto-generated).

For the native MATLAB implementation, the amount of time required to prepare one run for processing was recorded and multiplied by the number of runs, as PhysIO natively requires one configuration file to be created for each run. It can also leverage the SPM batch GUI, but this also requires a separate configuration step for each run. The same estimate calculation was done for computation time. For the CBRAIN implementation, which is capable of batch processing files using a single configuration (Figure 4), the setup time was recorded as well as the total time from task execution to task completion for computation time.

## 3. Results

We compared the functionality, user time, and computation time involved in running a dataset of 62 subjects on CBRAIN and using the native MATLAB implementation of PhysIO. These results are summarized in Table 1.

**Table 1:** Benchmarking and feature comparison for 62 fMRI runs processed by PhysIO on CBRAIN and with a local MATLAB installation.

Implementation	MATLAB	CBRAIN
Operating system	Windows, MacOS, Linux	Any
Required software	MATLAB with SPM12	Web browser
Hard drive space	~10 GB	None
MATLAB License required	Yes	No
Setup time	Approximately 95 min (2–5 min for initial setup; 1–2 min per run). May be reduced using custom scripts.	5–10 min. Requires generation of file list for batch processing.
Computation time	Approximately 45 min (~45 s per run). Will depend on CPU speed and image resolution. See caption for CPU used.	E.g. 25 min, with 57 s of CPU time per run (executed in parallel). Includes queue time, and may depend on server demand. Executed remotely, therefore the user’s local machine is not being used for computation.
GUI	In SPM	Yes
Batch processing	No	Yes
Support for BIDS subject folders as input	No	Yes
Produces corrected image	No (Outputs can be used as regressors in a GLM in SPM)	Yes
Visualization of results	In SPM	Yes

Assumes an experienced user in all cases. The MATLAB version was run on an MSI computer running Windows 10 with an Intel i7-8565U CPU @ 1.80GHz.

### 3.1. User time

As a result of the addition of a batch processing feature, user time was greatly reduced for the CBRAIN case compared to the native MATLAB implementation. Setup time was estimated for the latter two as taking 2–5 min to configure and execute an initial run, with approximately 2 min required for every subsequent run, resulting in an average estimate of 95 min for the dataset of 62 runs. In CBRAIN, creation of the CBRAIN File List (CBCSV) increased setup time slightly, but only one task configuration was required, reducing setup time to 5–10 min.

**Figure 5:**
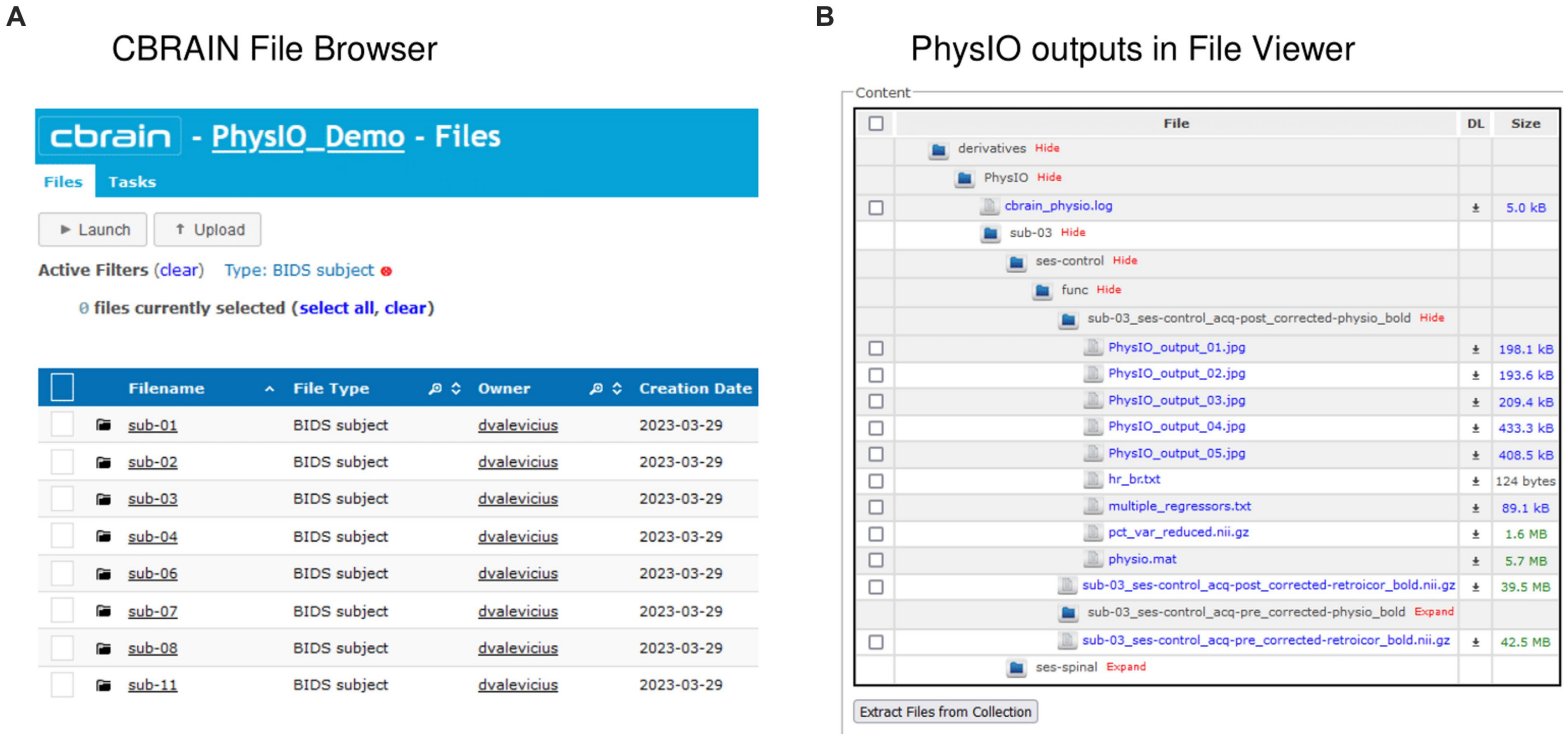
Input and output data representation in CBRAIN. **(A)** CBRAIN File Browser interface with data stored in BIDS Subject folders. **(B)** CBRAIN File Viewer window showing the contents of a PhysIO output folder.

An important feature of CBRAIN is provenance tracking of slight modifications to the tool parametrization. It enables users to save, and recall the parameter used for each command execution. As such, it facilitates reproducing results, and provides an easy and GUI-based interface to modify options, without risking deletion or overriding of data processed with different options. All these logs, as well as reports of task completion and failure are available to users on the interface (see Figure 5 for examples of file browsing and output file viewing in CBRAIN).

### 3.2. Computation time

On the locally installed softwares, using the RETROICOR modeling algorithm and an fMRI run with dimensions 80 × 80 × 40 × 190, a PhysIO task took 45 s to complete. With 62 runs, this resulted in an overall computation time of approximately 45 min.

On CBRAIN, computation time could be reduced by leveraging the batch processing functionality native to CBRAIN. The 62 runs could be processed in parallel across multiple HPC clusters. Thus, despite queue times, all tasks were completed in approximately 25 min. Longer queue times, however, would result in longer time-to-completion. The total time required to execute the task on a cluster (walltime) was 57 s. Thus, the lower bound for processing a batch of runs with PhysIO on CBRAIN (assuming no queue) would be about 1 min. It also includes the extra computation step of the automated image correction module, described below, explaining why the processing time was slightly longer than the local run, which did not perform image correction.

### 3.3. Functionality

The functionality of the PhysIO toolbox was expanded in three main ways. First, an automated image correction module was added via a MATLAB wrapper. This module uses the multiple regressors outputted by PhysIO to model noise in an fMRI run. The model residuals are then extracted and saved as a noise-corrected fMRI image, which can be used to assess model performance. At present, we have not modified the PhysIO’s original parameterization, and therefore our improvements are only related to ease of data management work-flow, provenance tracking, and visualization. A future update to the PhysIO wrapper would also include the preprocessing steps of prewhitening and high-pass filtering, which avoid some pitfalls of physiological noise correction, and ensure validity of statistical testing performed on the output image (Bright et al., 2017; Chen et al., 2017).

Next, we adapted PhysIO to accept a BIDS-format dataset as input. Most data on CBRAIN are stored according to the community conventions known as the Brain Imaging Data Structure (BIDS). In addition, tools such as a BIDS converter prototype are available to help users to refactor data according to BIDS standards. With the option to use a BIDS subject or dataset as input, the amount of files or directories needing to be tracked is greatly reduced, as a BIDS subject typically contains multiple runs collected across one or more scanning sessions.

We also enabled an option for batch processing tasks in parallel. On CBRAIN, one task configuration, which is created and saved in the CBRAIN GUI, can execute a task on the full set of fMRI runs. These runs are processed in parallel across multiple HPC clusters. In the native MATLAB implementation, custom scripts would be required for batch processing using a single configuration file, or parallel processing across multiple CPU cores.

Finally, the CBRAIN provides multiple ease-of-use improvements, including a user-friendly GUI for setting task parameters and an online data visualizer. The task configuration interface provides descriptions and information about each argument, and the integration with the Boutiques framework for command-line description allowed us to include constraints and interdependencies between parameters. The NIfTI visualization tool is accessible directly in-browser when viewing files on CBRAIN. Once a task is completed, users can browse through its outputs and view any slice or frame of an fMRI run or other MRI-based data with a .nii extension.

## 4. Conclusion

### 4.1. Summary

In this case study, we provide a methodology for integrating complex, multimodal fMRI pipelines such as TAPAS PhysIO (Kasper et al., 2017; Frässle et al., 2021) onto CBRAIN (Sherif et al., 2014). The CBRAIN platform is a web-based, open-science infrastructure for facilitating large-scale integrative neuroimaging research, and is designed with FAIR principles in mind (Poline et al., 2022). Our choice of the PhysIO toolbox for prototyping the implementation of MATLAB-based fMRI tools was motivated by (a) the comprehensive modeling options of PhysIO, which provides users a single tool to integrate physiological data gathered during an fMRI scan, irrespective of the manufacturer, (b) it being an open-source pipeline, and (c) the importance to neuroimaging research of considering physiological modulation of the BOLD signal, and the fact that the topography of physiologically-correlated BOLD modulations is not random and may contain important information (Khalili-Mahani et al., 2013).

In addition to making PhysIO available through CBRAIN, we have added several quality-of-life features, such as a graphical user interface, BIDS formatting tool, computation of noise-variance maps that enable users to quickly compare the impact of various noise modeling options on the data, a linear regression model that performs voxel-wise correction of the fMRI data based on noise parameter estimates, and importantly, the ability to visualize the results online. These features significantly improve workflow in collaborative studies by enabling researchers to test numerous physiological correction models and perform quality assurance tests without having to move large volumes of data to local computers for visualization.

The integration of PhysIO was also intended to serve as a template for future integrations of MATLAB-based neuroimaging tools into CBRAIN. Using the integration pipeline described in this paper, additional software tools can be packaged for use on CBRAIN on an *ad-hoc* basis. This can be done in collaboration between CBRAIN development personnel and members of the neuroimaging research community, thereby serving the needs of researchers as they arise and widening the array of preprocessing and analysis options available to CBRAIN users. This would save research laboratories many hours of intensive work installing, scripting, and debugging local MATLAB tools, as well as improving the transparency and reproducibility of these processing steps. This collaborative, open-science ecosystem has the potential to greatly improve the accessibility, pace, and reproducibility of neuroimaging research.

### 4.2. Limitations and future directions

Our benchmarking experiments are limited. On a small fMRI dataset (N = 62) from a single manufacturer and a personal computer (typically available to a student), we have shown a nine-fold improvement in user setup time (10 min on CBRAIN versus 95 min on local computer), as well as improvements in processing time compared to the original MATLAB implementation. A more comprehensive benchmarking experiment should compare the efficiency of analyzing a larger dataset on the CBRAIN implementation of PhysIO versus running it on a network installation of MATLAB. The latter was not available to us. Finally, future studies should leverage this platform to evaluate the impact of applying different noise models on results of noise-sensitive RSfMRI metrics such as regional homogeneity (REHO) or fractional amplitude of low-frequence fluctuations (fALFF).

Further, while the BIDS converter provided by the CBRAIN implementation of PhysIO allows for the read-in and renaming of files and input data according to BIDS file naming convention, it does not allow for the restructuring and conversion of the data provided in non-BIDS format, such as .log files directly extracted from the scanner. A BIDS converter tool that can write and output into BIDS format will be part of a future PhysIO release.

Finally, an important future addition would be the capacity to perform prewhitening and high-pass filtering in conjunction with noise modeling when performing the physiological noise correction step. These preprocessing steps are important if future use of the corrected image for statistical analysis is desired, including statistical tests of model fit, since they remove spurious autocorrelations (Bright et al., 2017; Chen et al., 2017). In addition, combining these models with task-related regressors in a GLM would prevent task-related signal that correlates with physiological models from being removed during the noise correction step.

We have shown that the web-based implementation of PhysIO can dramatically increase the speed and ease of physiological image correction, decreasing the tool’s learning curve, as well as improving the accessibility, reproducibility, and interoperability of this pipeline. Broader CBRAIN-integration of state-of-the-art fMRI processing and analysis tools has the potential to accelerate the pace and quality of integrative brain imaging research. In future work, we will demonstrate the advantage of leveraging CBRAIN to run fMRI preprocessing through different analytical tools (such as FSL, fMRIprep, and SPM).

## Data availability statement

The original contributions presented in the study are included in the article/supplementary material, further inquiries can be directed to the corresponding author.

## Ethics statement

Ethical approval was not required for the study involving humans in accordance with the local legislation and institutional requirements. Written informed consent to participate in this study was not required from the participants or the participants’ legal guardians/next of kin in accordance with the national legislation and the institutional requirements.

## Author contributions

LK developed and provided open-access to PhysIO. DV and NK-M designed and led the integration of PhysIO onto CBRAIN. DV, NB, SB, and PR contributed to the implementation of PhysIO in the CBRAIN platform. RA, BC, and NK-M coordinated different aspects of the projects. NK-M and AE wrote the grant to support the projects. DV, NK-M, and JB wrote the manuscript. All authors contributed to the article and approved the submitted version.
